# The lancet commission obesity categories, excess adiposity, and BMI discordance in urban Chinese adults: a cross-sectional survey in Beijing

**DOI:** 10.3389/fnut.2026.1883408

**Published:** 2026-08-04

**Authors:** Zhenyu Liu, Wendong Wang, Wendong Cao, Qing Yao, Qi Zhou

**Affiliations:** 1 Department of Chronic Disease Prevention and Control, Haidian District Center for Disease Control and Prevention, Beijing, China; 2 Beijing Anzhen Hospital, Beijing Institute of Heart, Lung, and Blood Vessel Diseases, Capital Medical University, Beijing, China

**Keywords:** body mass index, Chinese adults, chronic disease surveillance, clinical obesity, excess adiposity, nutritional epidemiology, waist-to-height ratio

## Abstract

**Introduction:**

BMI remains central to obesity surveillance, but it does not distinguish excess adiposity from obesity-related disease. The Lancet Commission framework first confirms excess adiposity and then classifies individuals as having no obesity, preclinical obesity, or clinical obesity. We applied this framework using China-specific anthropometric cut points to estimate Lancet obesity categories, characterize clinical obesity, assess discordance with BMI categories, and examine related demographic and lifestyle factors in urban Chinese adults.

**Methods:**

We analyzed data from the 2024 Haidian Chronic Disease and Risk Factor Surveillance, a stratified multistage probability cluster survey in urban Beijing. Excess adiposity was confirmed using BMI, waist circumference, and waist-to-height ratio. Lancet obesity categories were assigned after assessment of obesity-related complications. Survey-weighted prevalence estimates and ordinal logistic regression were used.

**Results:**

The final analytic sample included 8,130 adults aged 18–79 years. The weighted prevalence of excess adiposity was 41.2%, and the prevalence of clinical and preclinical obesity was 37.3% and 3.9%, respectively. The most frequent complications among participants with clinical obesity were metabolic impairment and hypertension. BMI categories were substantially discordant with Lancet obesity categories: clinical obesity was present in 5.3% of participants with BMI <24 kg/m^2^ and 41.6% of those with BMI 24.0–27.9 kg/m^2^, whereas 3.4% of participants with BMI ≥28 kg/m^2^ had no obesity. Female sex was associated with lower odds of being in a higher ordered Lancet obesity category (OR, 0.46; 95% CI, 0.40–0.53).

**Discussion:**

A substantial clinical obesity burden and marked discordance with BMI categories were identified in urban Beijing adults when applying the Lancet framework. These findings support complementing BMI with assessment of excess adiposity and obesity-related complications in chronic disease surveillance.

## Introduction

Obesity has emerged as a dominant driver of chronic disease burden worldwide, with pooled analyses documenting consistent increases in prevalence across children, adolescents, and adults between 1990 and 2022 (1). In China, rapid nutrition transition, accelerating urbanization, and population aging have been accompanied by parallel rises in overweight, obesity, abdominal adiposity, and related metabolic disease (2–5). Among urban adults in particular, excess adiposity frequently coexists with hypertension, dysglycaemia, dyslipidaemia, and renal impairment. Effective population surveillance must therefore detect not only excess body weight but also the presence or absence of accompanying clinical or organ-level functional impairment.

Body mass index (BMI) remains the most widely used adiposity measure owing to its simplicity, low cost, and reproducibility. However, BMI cannot distinguish fat mass from lean mass or capture central fat distribution, and adiposity-related risk varies substantially by sex, age, and ethnicity (6, 7). These limitations are especially relevant in Asian populations, in whom cardiometabolic risk emerges at BMI levels below conventional European thresholds (8, 9). Central adiposity indices such as the waist-to-height ratio (WHtR) provide prognostic information beyond BMI for hypertension and broader cardiometabolic risk (10, 11). Sole reliance on BMI may therefore underestimate risk in individuals with normal or near-normal BMI but significant central adiposity, while potentially misclassifying those with elevated BMI but no objective clinical or organ-level impairment as obese.

Recent frameworks have shifted from defining obesity by body size alone to defining it as a disease state grounded in excess adiposity and consequent organ dysfunction. The European Association for the Study of Obesity (EASO) proposed a framework integrating anthropometric measures with medical, functional, and psychological complications (12). In 2025, the Lancet Diabetes & Endocrinology Commission advanced operational diagnostic criteria: excess adiposity is first confirmed by anthropometric measures, and individuals are then stratified into Preclinical obesity (excess adiposity without organ dysfunction) or Clinical obesity (excess adiposity with objective organ dysfunction or functional limitation) (13). Translating this framework to Chinese populations requires the use of China-specific BMI and waist circumference thresholds.

This diagnostic framework carries important implications for population surveillance, clinical risk stratification, and the prioritization of prevention strategies, yet its performance in routine chronic disease surveillance among urban Chinese adults, using China-specific anthropometric thresholds, has not been well characterized. Using data from the 2024 Haidian Chronic Disease and Risk Factor Surveillance (HCDRFS), we applied the Lancet Commission definition of obesity through a two-step framework with four objectives: (1) to estimate the weighted prevalence of excess adiposity and Lancet obesity categories, including no obesity, preclinical obesity, and clinical obesity; (2) to characterize the complication profile of Clinical obesity; (3) to quantify discordance between Lancet obesity categories and BMI categories; and (4) to identify demographic and lifestyle correlates of the ordered Lancet obesity category. These analyses aim to establish the epidemiological utility of this disease-oriented obesity definition and to guide its interpretation in urban Chinese adult populations. By linking anthropometric adiposity measures with metabolic and organ-function indicators, this study also addresses nutrition-related chronic disease surveillance in the context of China's rapid nutrition transition.

## Materials and methods

### Study design and participants

**Figure 1:**
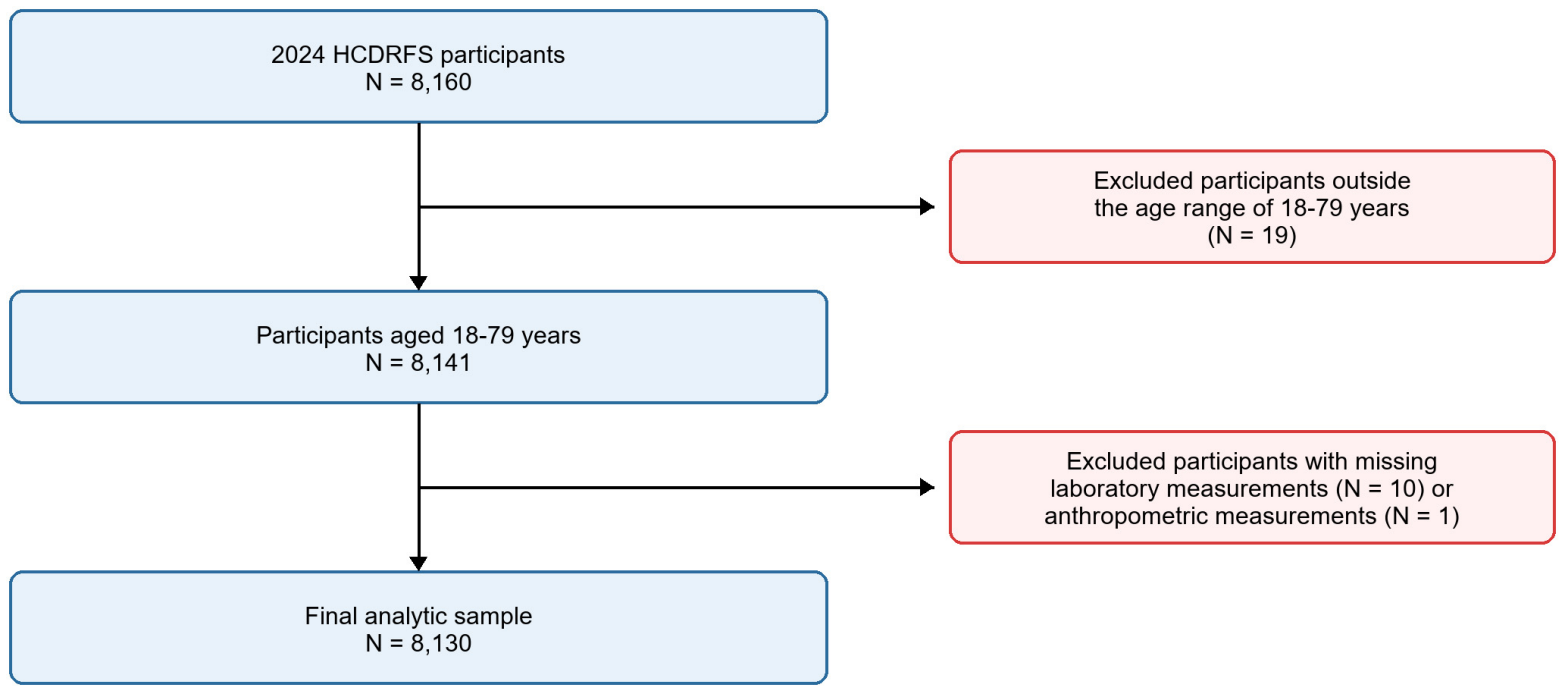
Flowchart of participant selection for the analytic sample. HCDRFS, Haidian Chronic Disease and Risk Factor Surveillance.

The 2024 Haidian Chronic Disease and Risk Factor Surveillance (HCDRFS 2024) was a cross-sectional survey covering all 29 townships in Haidian District, Beijing, conducted by the Haidian District Center for Disease Control and Prevention from June 2023 to October 2024. HCDRFS 2024 used a stratified multistage probability cluster sampling design to generate a regionally representative sample. In the first stage, four communities or villages were selected using probability-proportional-to-size (PPS) sampling. In the second stage, each community or village was divided into household groups of at least 70 households, and one household group was randomly selected from each sampling unit. Finally, one participant aged 18 to 79 years was selected from each household using the Kish grid method. Inclusion criteria were: (1) age ≥18 years; (2) residence at the current address for more than 6 months during the previous 12 months; (3) not pregnant; and (4) absence of any severe health condition or disease that might prevent participation, including intellectual disability or language impairment. The target sample size was 8,120, and 8,160 participants were recruited from 116 communities or villages. We excluded participants outside the age range of 18 to 79 years (*n* = 19), those with missing laboratory tests (*n* = 10), and those with missing anthropometric measurements (*n* = 1). The final analytic sample included 8,130 adults (4,216 men and 3,914 women). The study flowchart is presented in Figure 1, and the operational classification algorithm is presented in Supplementary Figure S1.

The study protocol was approved by the Ethics Review Committee of Beijing Zhongguancun Hospital (approval No. 20240325-WangWD; approval date: 25 March 2024), and all participants provided written informed consent. The study followed the Strengthening the Reporting of Observational Studies in Epidemiology (STROBE) reporting guideline (14).

### Data collection

All study personnel received standardized training. Sociodemographic and lifestyle information was collected through face-to-face interviews, covering sociodemographic characteristics, behavioral and dietary habits, physical activity, and personal and family disease history. Physical examinations included height, weight, waist circumference, blood pressure, and pulse. Body weight was measured with an electronic scale accurate to 0.1 kg (Tanita HD394, Beijing, China), and height was measured with a mechanical stadiometer accurate to 0.1 cm (TZG, Wuxi, China). Waist circumference was measured with the participant in a standing position, at the midpoint between the lower margin of the costal arch and the upper margin of the iliac crest. For blood pressure, participants rested in a seated position for 5 min; blood pressure was then measured three times consecutively on the non-dominant arm using an automated electronic blood pressure monitor (Omron HBP-1300, Kyoto, Japan), with a 1-min interval between measurements. The mean of the three readings was used in subsequent analyses.

### Laboratory measurements

Venous blood samples of 7.5 ml were collected from all participants after at least 10 hours of fasting to measure fasting blood glucose (FBG), glycated hemoglobin (HbA1c), total cholesterol (TC), low-density lipoprotein cholesterol (LDL-C), high-density lipoprotein cholesterol (HDL-C), triglycerides (TG), blood uric acid (BUA), and serum creatinine (Scr). Samples were centrifuged and aliquoted at the survey site and were stored and transported according to protocol. Morning midstream urine samples of 15 ml were collected to measure urinary creatinine (UCr) and urine microalbumin (UMA).

Laboratory tests were performed centrally by a third-party laboratory. FBG, TC, LDL-C, HDL-C, TG, BUA, Scr, and urinary biochemical indicators were measured on a Beckman AU680 automated biochemical analyser. FBG was measured by the hexokinase method, TC by the cholesterol oxidase method, LDL-C and HDL-C by direct methods, TG by the glycerol phosphate oxidase method, BUA by the uricase ultraviolet method, Scr and UCr by enzymatic methods, and UMA by immunoturbidimetry. HbA1c was measured on a Hangzhou Huizhong MQ-6000 hemoglobin analyser using high-performance liquid chromatography.

### Definition of excess adiposity and lancet obesity categories

Following the Lancet Diabetes & Endocrinology Commission guideline, excess adiposity was operationally defined using BMI, waist circumference, and WHtR, with China-specific cut-offs for BMI and waist circumference and the commonly used threshold of 0.50 for WHtR (15, 16). Participants were considered to have excess adiposity if they met any of the following criteria: BMI ≥40.0 kg/m^2^; 28.0 ≤ BMI < 40.0 kg/m^2^ with elevated waist circumference (≥ 90 cm in men, ≥85 cm in women); 28.0 ≤ BMI < 40.0 kg/m^2^ with WHtR ≥0.50; or BMI < 28.0 kg/m^2^ with both elevated waist circumference and WHtR ≥0.50. Among participants with excess adiposity, obesity status was further stratified by the presence or absence of obesity-related organ dysfunction as follows: preclinical obesity, defined as excess adiposity without objective organ dysfunction; and clinical obesity, defined as excess adiposity with objective organ dysfunction or functional limitation. The obesity-related organ dysfunctions and functional limitations used for classification included metabolic impairment, hypertension, chronic kidney disease, chronic respiratory disease, musculoskeletal disease, coronary heart disease, and atrial fibrillation; their operational definitions are provided in Supplementary Figure S1.

### Covariates

Sociodemographic and lifestyle characteristics and related risk factors were self-reported during face-to-face interviews. Variables included age, sex, marital status (married or unmarried), education (primary school or below, junior high school, senior high school, and college or above), smoking status (nonsmoker, former smoker, or current smoker), excessive alcohol consumption (alcohol consumption of 15 g/d or greater for women and 25 g/d or greater for men), low physical activity (< 150 minutes/week) (17), insufficient fruit and vegetable intake (< 400 g/d), excess red meat intake (≥100 g/d) (18), sleep duration ( ≤ 7 h, 7 to 9 h, and ≥9 h) (19), and insomnia [defined as an Insomnia Severity Index (ISI) score ≥10] (20).

### Statistical analysis

All analyses accounted for the complex survey design, including stratification, clustering, and sampling weights. Sampling weights were the product of the original sampling weights, non-response weights, and post-stratification weights. Post-stratification weights were calibrated to the 2020 seventh national population census for residents of Haidian District, Beijing. Continuous variables are expressed as weighted means ± weighted standard deviations, skewed variables as medians (interquartile ranges, IQRs), and categorical variables as unweighted frequencies and weighted proportions. Between-group differences were tested using survey-weighted linear regression with Wald tests for normally distributed continuous variables, survey-weighted quantile regression for skewed continuous variables, and Rao-Scott chi-square tests for categorical variables. Because the proportion of participants with missing data was very small (< 0.4%), complete-case analysis was performed without imputation. Survey-weighted ordered logistic regression models (proportional odds models) were fitted with the ordered Lancet obesity category (no obesity, preclinical obesity, and clinical obesity) as the dependent variable and sociodemographic factors (age, sex, marital status, and education) and lifestyle factors (smoking, excessive alcohol consumption, low physical activity, insufficient fruit and vegetable intake, excess red meat intake, and sleep duration) as independent variables. Cumulative odds ratios (ORs) with 95% confidence intervals (CIs) were reported. The proportional odds assumption was assessed graphically and by comparing log-odds ratios across binary cut points and was considered acceptable for the final model. All analyses were performed in R version 4.5.0 (R Foundation for Statistical Computing, Vienna, Austria) using the survey package. A two-sided *P* value < 0.05 was considered statistically significant.

## Results

### Baseline characteristics

The final analytic sample included 8,130 adults aged 18–79 years. Of these, 4,216 were men and 3,914 were women. Statistically significant sex differences were observed in age, marital status, education, smoking, alcohol consumption, dietary indicators, insomnia, BMI, waist circumference, blood pressure, fasting glucose, triglycerides, HDL-C, LDL-C, and eGFR (Table 1). Baseline characteristics by age group are shown in Supplementary Table S1.

**Table 1:** Baseline characteristics of participants by sex.

Variable	Men	Women	*P* value
Number (%)	4,216 (49.7)	3,914 (50.3)	
Age, years	42.2 ± 15.5	43.1 ± 15.8	0.003
Marital status			0.019
Unmarried	1,009 (31.7)	908 (28.5)	
Married	3,207 (68.3)	3,006 (71.5)	
Education level			< 0.001
Primary school or below	226 (4.3)	272 (6.0)	
Junior high school	1,224 (26.0)	708 (15.8)	
Senior high school	1,058 (25.1)	835 (21.0)	
College or above	1,708 (44.6)	2,099 (57.2)	
Smoking status			< 0.001
nonsmoker	2,185 (53.0)	3,765 (96.3)	
former smoker	240 (4.9)	25 (0.7)	
current smoker	1,791 (42.1)	124 (3.0)	
Excessive alcohol consumption			< 0.001
No	3,705 (89.1)	3,858 (98.6)	
Yes	511 (10.9)	56 (1.4)	
Low physical activity			0.497
No	3,120 (73.4)	2,920 (74.2)	
Yes	1,096 (26.6)	994 (25.8)	
Insufficient fruit and vegetable intake			< 0.001
No	2,352 (54.0)	2,564 (64.2)	
Yes	1,864 (46.0)	1,350 (35.8)	
Excess red meat intake			< 0.001
No	2,787 (63.9)	2,978 (74.8)	
Yes	1,429 (36.1)	936 (25.2)	
Height, cm	171.9 ± 6.6	160.3 ± 6.1	< 0.001
Weight, kg	77.2 ± 14.3	62.4 ± 10.8	< 0.001
BMI, kg/m^2^	26.1 ± 4.2	24.3 ± 4.0	< 0.001
Waist circumference, cm	90.0 ± 11.2	79.4 ± 10.4	< 0.001
WHtR	0.5 ± 0.1	0.5 ± 0.1	< 0.001
Total sleep duration			0.337
≤ 7 h	558 (12.5)	471 (11.7)	
7~9 h	2,039 (47.8)	1,963 (50.2)	
≥9 h	1,619 (39.7)	1,480 (38.1)	
Insomnia			< 0.001
No	3,927 (93.4)	3,511 (89.6)	
Yes	289 (6.6)	403 (10.4)	
BMI category			< 0.001
Underweight	74 (2.3)	157 (4.1)	
Normal	1,232 (29.8)	1,832 (47.4)	
Overweight	1,732 (39.9)	1,251 (31.0)	
Obesity	1,178 (28.1)	674 (17.5)	
Lancet obesity category			< 0.001
No obesity	1,927 (48.9)	2,632 (68.6)	
Preclinical obesity	211 (5.3)	93 (2.6)	
Clinical obesity	2,078 (45.8)	1,189 (28.8)	
Diagnostic components for clinical obesity			
Metabolic impairment			0.001
No	271 (12.9)	109 (7.8)	
Yes	1,807 (87.1)	1,080 (92.2)	
Hyperglycemia			0.337
No	1,168 (58.8)	670 (56.4)	
Yes	910 (41.2)	519 (43.6)	
Hypertriglyceridemia			0.001
No	751 (35.1)	586 (48.9)	
Yes	1,327 (64.9)	603 (51.1)	
Low HDL-C			< 0.001
No	1,145 (51.1)	397 (32.7)	
Yes	933 (48.9)	792 (67.3)	
Hypertension			0.001
No	954 (45.8)	658 (57.3)	
Yes	1,124 (54.2)	531 (42.7)	
Chronic kidney disease			0.346
No	1,689 (82.3)	1,006 (84.0)	
Yes	389 (17.7)	183 (16.0)	
Chronic respiratory disease			0.030
No	1,979 (95.5)	1,119 (93.0)	
Yes	99 (4.5)	70 (7.0)	
Musculoskeletal disease			< 0.001
No	1,594 (78.5)	736 (61.9)	
Yes	484 (21.5)	453 (38.1)	
Coronary heart disease			0.583
No	1,980 (96.3)	1,155 (96.9)	
Yes	98 (3.7)	34 (3.1)	
Atrial fibrillation			0.438
No	2,048 (98.7)	1,173 (98.2)	
Yes	30 (1.3)	16 (1.8)	
BP and biochemical parameters			
SBP, mm Hg	137.1 ± 16.5	133.1 ± 18.3	< 0.001
DBP, mm Hg	85.2 ± 11.8	78.0 ± 10.7	< 0.001
Fasting glucose, mmol/L	6.1 ± 2.4	5.8 ± 1.6	0.001
HbA1c, %	6.1 ± 1.2	6.0 ± 0.9	0.275
Total cholesterol, mmol/L	4.9 ± 1.0	5.0 ± 1.1	0.177
Triglycerides, mmol/L	2.7 ± 2.9	1.8 ± 1.0	< 0.001
HDL-C, mmol/L	1.0 ± 0.2	1.2 ± 0.3	< 0.001
LDL-C, mmol/L	3.0 ± 0.8	3.1 ± 0.8	0.035
UACR, mg/g	4.5 (0.2, 9.5)	5.5 (0.2, 11.0)	0.102
eGFR, mL/min/1.73 m^2^	103.7 ± 17.7	101.2 ± 17.9	0.004

Values are presented as unweighted number (weighted %), weighted mean ± weighted SD, or median (IQR). *P* values compare men and women using survey-weighted tests. BMI, body mass index; WHtR, waist-to-height ratio; SBP, systolic blood pressure; DBP, diastolic blood pressure; HbA1c, glycated hemoglobin; HDL-C, high-density lipoprotein cholesterol; LDL-C, low-density lipoprotein cholesterol; UACR, urinary albumin-to-creatinine ratio; eGFR, estimated glomerular filtration rate; ISI, Insomnia Severity Index.

### Prevalence and diagnostic composition of excess adiposity

Overall, 3,571 participants met the excess-adiposity criteria, corresponding to a weighted prevalence of 41.2% (Table 2). The prevalence was higher in men than in women (51.1% vs. 31.4%; *P* < 0.001). The largest diagnostic components were 28.0 ≤ BMI < 40.0 kg/m^2^ with elevated waist circumference and BMI < 28.0 kg/m^2^ with both elevated waist circumference and WHtR ≥0.50, accounting for 48.4% and 46.7% of participants with excess adiposity, respectively. The weighted prevalence was 37.3% for clinical obesity and 3.9% for preclinical obesity.

**Table 2:** Distribution of excess adiposity criteria and Lancet obesity categories overall and by sex.

Indicator	Overall	Women	Men	*P* value
Participants, *n*	8,130	3,914	4,216	
Excess adiposity criteria				
BMI ≥40.0 kg/m^2^	42 (1.3)	9 (0.6)	33 (1.6)	0.040
28.0 ≤ BMI < 40.0 kg/m^2^ with elevated waist circumference	1,677 (48.4)	588 (45.8)	1,089 (50.1)	0.059
28.0 ≤ BMI < 40.0 kg/m^2^ with WHtR ≥0.50	90 (3.7)	54 (6.6)	36 (1.8)	< 0.001
BMI < 28.0 kg/m^2^ with both elevated waist circumference and WHtR ≥0.50	1,762 (46.7)	631 (47.0)	1,131 (46.4)	0.843
Total participants with excess adiposity	3,571 (41.2)	1,282 (31.4)	2,289 (51.1)	< 0.001
Lancet obesity category				< 0.001
No obesity (no excess adiposity)	4,559 (58.8)	2,632 (68.6)	1,927 (48.9)	
Preclinical obesity	304 (3.9)	93 (2.6)	211 (5.3)	
Clinical obesity	3,267 (37.3)	1,189 (28.8)	2,078 (45.8)	

Values are presented as unweighted number (weighted %). *P* values compare women and men. BMI, body mass index; WC, waist circumference; WHtR, waist-to-height ratio.

### Clinical obesity and obesity-related complications

**Figure 2:**
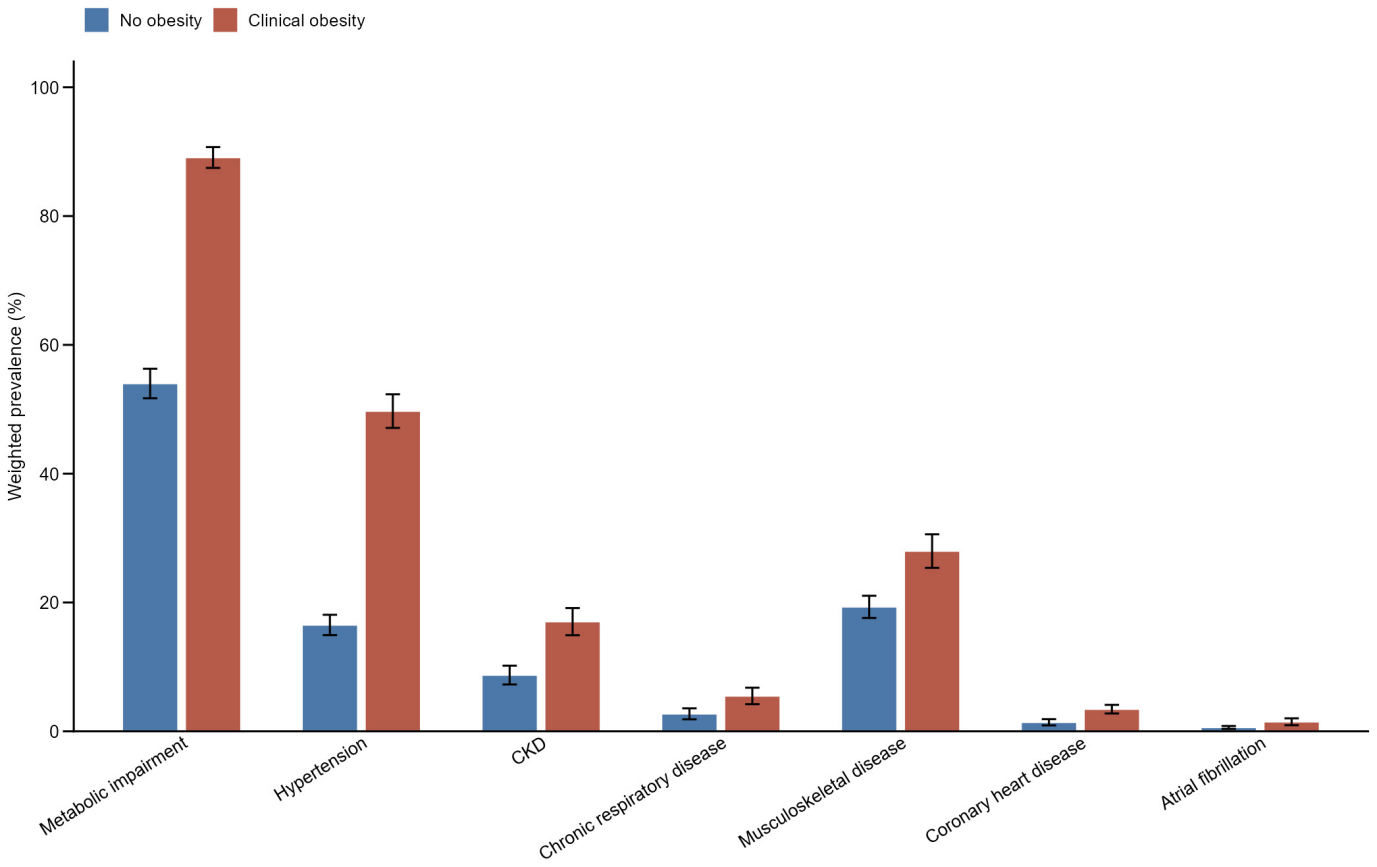
Survey-weighted prevalence of obesity-related complications among adults with no obesity and clinical obesity under the Lancet framework. Error bars indicate 95% confidence intervals. All estimates incorporate complex survey weights. CKD, chronic kidney disease; CHD, coronary heart disease.

Participants with clinical obesity had a higher weighted prevalence of all seven obesity-related complications than those with no obesity (Figure 2, *P* < 0.001 for all comparisons except chronic respiratory disease, P = 0.003). The largest differences were observed for metabolic impairment (89.1% vs. 54.0%) and hypertension (49.7% vs. 16.5%). By sex, women had higher prevalences of metabolic impairment, low HDL-C, musculoskeletal disease, and chronic respiratory disease than men, whereas men had higher prevalences of hypertriglyceridaemia and hypertension (Table 1).

### Concordance with BMI categories

**Figure 3:**
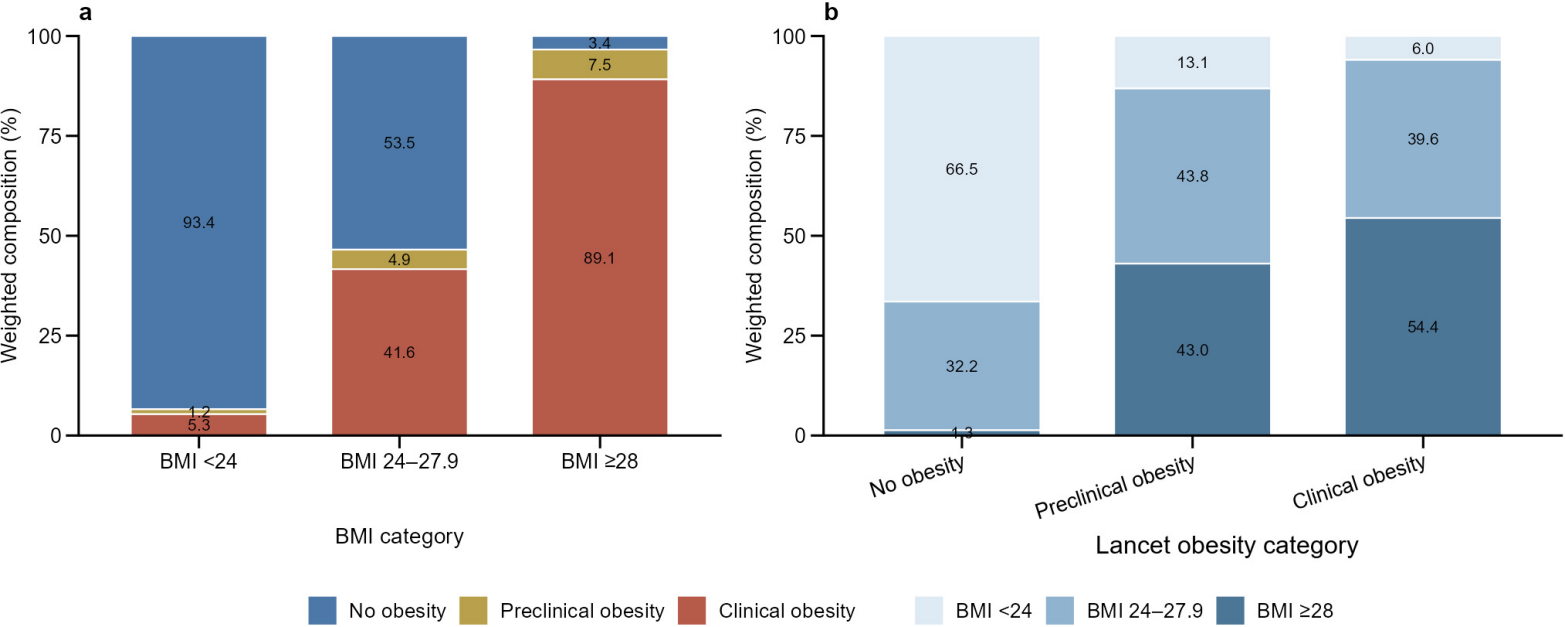
Discordance between BMI categories and Lancet obesity categories. **(a)** shows Lancet obesity categories within each BMI category; **(b)** shows BMI category distribution within each Lancet obesity category. All proportions are survey-weighted. BMI, body mass index.

BMI categories showed discordance with Lancet obesity categories (Figure 3; Supplementary Table S2). Among participants with Normal weight (BMI < 24.0 kg/m^2^), 5.3% were classified as having clinical obesity and 1.2% as having preclinical obesity. Among those with Overweight (BMI 24.0–27.9 kg/m^2^), 41.6% had clinical obesity and 4.9% had preclinical obesity. Among participants with Obesity (BMI ≥28.0 kg/m^2^), 89.1% had clinical obesity, 7.5% had preclinical obesity, and 3.4% had no obesity.

### Age- and sex-specific distribution of lancet obesity categories

**Figure 4:**
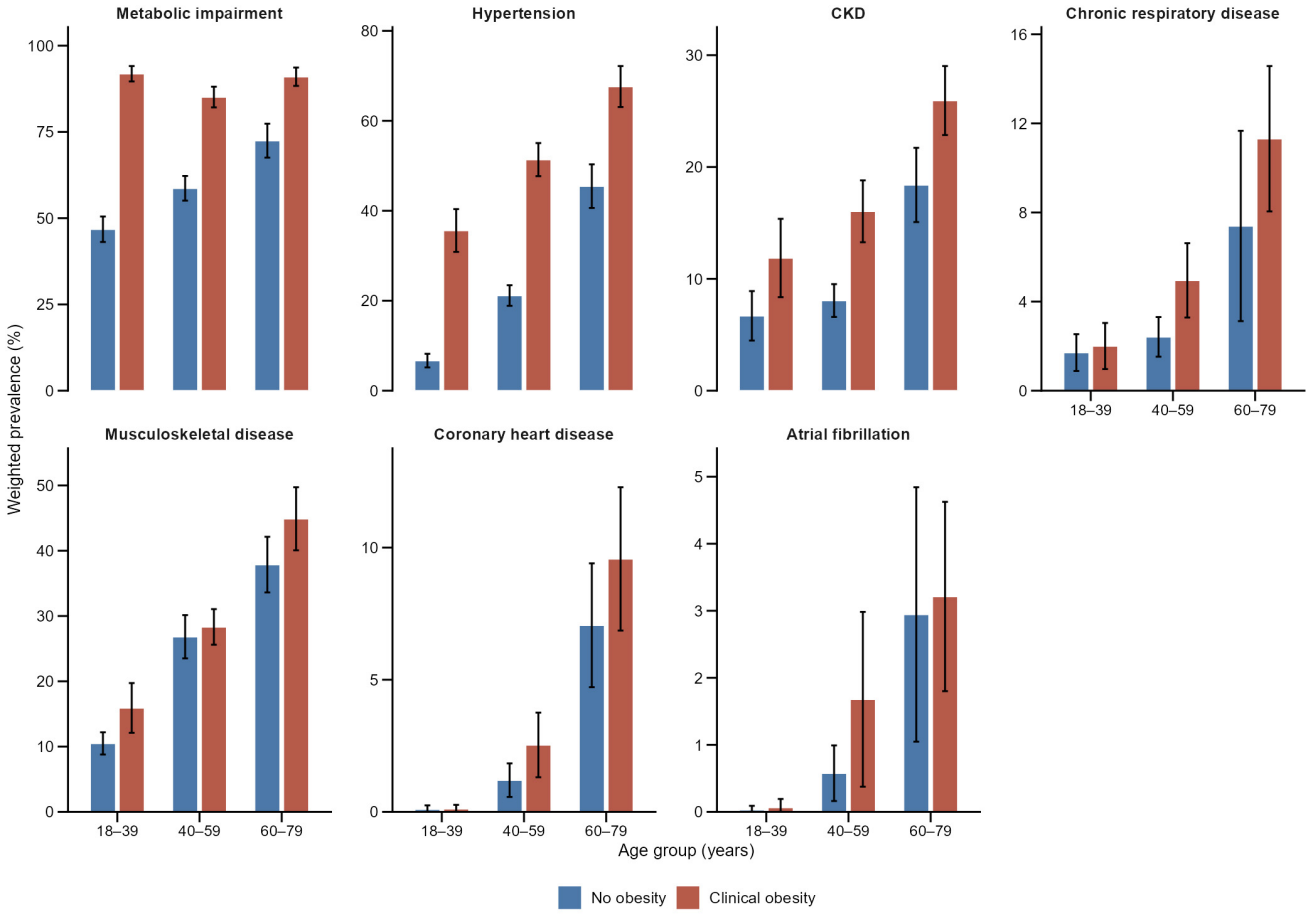
Age-specific patterns of Lancet obesity categories and obesity-related complications. Blue bars indicate no obesity; vermillion bars indicate clinical obesity. All estimates are survey-weighted.

The weighted prevalence of clinical obesity increased with age, from 28.2% in adults aged 18–39 years to 42.1% in those aged 40–59 years and 52.9% in those aged 60–79 years (Supplementary Table S3). The prevalence of preclinical obesity decreased across the same age groups, from 5.1% to 3.3% and 2.0%. Complication prevalence also increased with age, particularly for hypertension, chronic kidney disease, chronic respiratory disease, coronary heart disease, atrial fibrillation, and metabolic impairment (Figure 4).

### Correlates of the ordered lancet obesity category

Survey-weighted ordinal logistic regression results for the ordered Lancet obesity category are shown in Table 3. The proportional odds assumption was acceptable, with a maximum |ΔlogOR| of 0.128 (Supplementary Table S4). In the total population, female sex was associated with a lower ordered Lancet obesity category (OR, 0.46; 95% CI, 0.40–0.53). Age 40–59 years, age 60–79 years, being married, and current smoking were associated with a higher ordered Lancet obesity category. In men, age 40–59 years, being married, and current smoking were associated with a higher category, whereas insufficient fruit and vegetable intake and sleep duration ≥9 h were associated with a lower category. In women, age 40–59 years, age 60–79 years, and junior high school education were associated with a higher category.

**Table 3:** Survey-weighted ordinal logistic regression for the ordered Lancet obesity category.

Variable	Total OR (95% CI)	Total *P* value	Men OR (95% CI)	Men *P* value	Women OR (95% CI)	Women *P* value
Sex						
Men	Reference					
Women	0.46 (0.40–0.53)	< 0.001	—	—	—	—
Age group, years						
18–39	Reference		Reference		Reference	
40–59	1.52 (1.28–1.80)	< 0.001	1.36 (1.14–1.63)	< 0.001	1.71 (1.35–2.18)	< 0.001
60–79	2.46 (1.88–3.23)	< 0.001	1.30 (1.00–1.71)	0.054	3.92 (2.82–5.43)	< 0.001
Education						
Primary school or below	Reference	—	Reference	—	Reference	—
Junior high school	1.26 (0.97–1.64)	0.085	1.16 (0.80–1.67)	0.431	1.58 (1.06–2.36)	0.025
Senior high school	0.93 (0.73–1.19)	0.566	0.94 (0.61–1.44)	0.762	0.96 (0.67–1.38)	0.841
College or above	0.94 (0.76–1.16)	0.572	1.31 (0.90–1.90)	0.165	0.73 (0.53–1.01)	0.056
Marital status						
Unmarried	Reference	—	Reference	—	Reference	—
Married	1.23 (1.05–1.43)	0.009	1.58 (1.28–1.95)	< 0.001	0.99 (0.80–1.22)	0.938
Smoking status						
Nonsmoker	Reference	—	Reference	—	Reference	—
Former smoker	0.82 (0.58–1.16)	0.259	0.90 (0.63–1.28)	0.550	1.17 (0.45–3.06)	0.749
Current smoker	1.15 (1.01–1.31)	0.041	1.18 (1.03–1.37)	0.021	1.16 (0.69–1.96)	0.572
Excessive alcohol consumption						
No	Reference	—	Reference	—	Reference	—
Yes	1.20 (0.92–1.56)	0.173	1.24 (0.97–1.59)	0.083	1.42 (0.52–3.90)	0.491
Low physical activity						
No	Reference	—	Reference	—	Reference	—
Yes	0.99 (0.88–1.12)	0.901	1.02 (0.86–1.20)	0.830	1.01 (0.79–1.30)	0.928
Insufficient fruit and vegetable intake						
No	Reference	—	Reference	—	Reference	—
Yes	0.90 (0.79–1.03)	0.122	0.83 (0.70–0.99)	0.039	0.97 (0.76–1.24)	0.783
Excess red meat intake						
No	Reference	—	Reference	—	Reference	—
Yes	1.01 (0.90–1.13)	0.886	0.90 (0.77–1.05)	0.194	1.15 (0.92–1.44)	0.214
Sleep duration						
≤ 7 h	1.07 (0.88–1.31)	0.483	1.17 (0.92–1.50)	0.198	0.99 (0.70–1.40)	0.953
7~9 h	Reference	—	Reference	—	Reference	—
≥9 h	0.89 (0.79–1.01)	0.065	0.82 (0.70–0.96)	0.012	1.01 (0.81–1.27)	0.897
Insomnia						
No	Reference	—	Reference	—	Reference	—
Yes	0.83 (0.65–1.06)	0.128	0.79 (0.56–1.10)	0.164	0.77 (0.56–1.06)	0.110

Survey-weighted ordinal logistic regression estimated cumulative odds ratios for the ordered outcome (no obesity < preclinical obesity < clinical obesity) under the proportional odds assumption. ORs are mutually adjusted for sex (total model only), age group, education, marital status, smoking status, excessive alcohol consumption, low physical activity, insufficient fruit and vegetable intake, excess red meat intake, sleep duration, and insomnia. Sex-stratified models exclude sex. All analyses incorporated complex survey weights. OR, odds ratio; CI, confidence interval.

## Discussion

In this surveillance-based study of urban Chinese adults, application of the Lancet Commission obesity framework identified a high burden of clinical obesity (37.3%) and a much smaller group with preclinical obesity (3.9%). Overall, 41.2% of participants met the criteria for excess adiposity. BMI categories were markedly discordant with Lancet obesity categories: clinical obesity was observed among participants with BMI < 24 kg/m^2^ and BMI 24.0–27.9 kg/m^2^, whereas some participants with BMI ≥28 kg/m^2^ were classified as having no obesity or preclinical obesity. These findings indicate that clinical obesity, under the Lancet Commission definition, is not equivalent to higher BMI, but represents a disease state characterized by excess adiposity with obesity-related organ dysfunction.

Our findings are consistent with recent applications of the Lancet framework in other populations. Studies from the All of Us cohort, Korean KNHANES, CNHS 2015, and Hong Kong community cohorts have similarly shown that disease-state definitions identify discordance between BMI categories and clinical status (21–24). The main point of convergence is not the absolute prevalence estimate, but the distinction between BMI-based screening categories and Lancet obesity categories. The Hong Kong cohorts further suggested that clinical obesity is relevant to long-term cardiovascular-kidney-metabolic risk stratification (24). However, cross-study comparisons require caution because anthropometric thresholds, complication definitions, functional measures, available variables, and population structure may differ across settings. In Asian populations, recent clinical obesity literature emphasizes the need for ethnicity-appropriate adiposity assessment and complication-based classification, rather than direct comparison of prevalence estimates across settings (9, 13, 24). Surveillance datasets may not capture the full spectrum of obesity-related complications, which can affect estimates of preclinical and clinical obesity.

The discordance between BMI categories and Lancet obesity categories reinforces the limitations of BMI as a disease-state classifier. BMI remains useful for screening, but it cannot capture fat distribution, visceral or ectopic adiposity, or metabolic heterogeneity (7, 9, 25, 26). Central adiposity measures such as WHtR add information beyond BMI and are useful for cardiometabolic risk screening in adults (11). Previous work on metabolically healthy obesity and metabolically unhealthy normal weight similarly shows that people with comparable BMI values can differ substantially in cardiometabolic risk (27–29). The Lancet framework does not replace BMI. Rather, it uses BMI and central adiposity measures to confirm excess adiposity before obesity-related organ dysfunction or functional limitation is considered.

The diagnostic composition of clinical obesity was dominated by metabolic impairment and hypertension. These conditions were present in 89.1% and 49.7% of participants with clinical obesity, compared with 54.0% and 16.5%, respectively, among participants with no obesity. Chronic kidney disease, musculoskeletal disease, chronic respiratory disease, coronary heart disease, and atrial fibrillation were also more frequent among participants with clinical obesity. Prior studies have linked high BMI, abdominal adiposity, and their combination with chronic disease and cardiovascular disease risk (30, 31). However, this comparison requires caution because obesity-related complications are part of the definition of clinical obesity. The higher prevalence of complications among participants with clinical obesity therefore partly reflects the classification rule itself. Accordingly, the complication analysis should be interpreted as describing diagnostic composition and classification drivers, not as evidence of independent associations between excess adiposity and individual complications.

The low proportion of preclinical obesity also warrants interpretation. Under the Lancet Commission framework, individuals with confirmed excess adiposity are classified as having clinical obesity when obesity-related organ dysfunction or clinically relevant functional limitation is present, and as having preclinical obesity when these features are absent (13). In this adult surveillance sample, many participants with excess adiposity also had metabolic impairment, hypertension, or another obesity-related complication. The small preclinical obesity group may therefore reflect the high background burden of measured cardiometabolic abnormalities and the complication-based structure of the classification. It should not be interpreted as evidence that early adiposity-related risk was uncommon. Age patterns were consistent with this interpretation. Clinical obesity increased with age, whereas preclinical obesity declined, as also reported in Korean KNHANES and CNHS 2015 analyses (22, 23). Sex differences were also observed: men had a higher overall burden of clinical obesity, whereas women had lower odds of being in a higher ordered Lancet obesity category. These differences should not be interpreted as causal effects of sex. They may reflect differences in fat distribution, waist circumference thresholds, age structure, menopause-related metabolic changes, health behaviors, healthcare use, and chronic disease diagnosis. Recent longitudinal and intervention studies suggest prognostic and risk-stratification relevance for preclinical and clinical obesity categories; however, in this cross-sectional study, the regression results should be interpreted as surveillance-based associations rather than determinants of future clinical obesity (32–34).

This study has limitations. First, the cross-sectional design cannot establish the temporal sequence between excess adiposity, lifestyle factors, and obesity-related organ dysfunction, nor determine whether preclinical obesity progresses to clinical obesity over time. Second, several complications were based on self-reported disease history or proxy definitions, which may have introduced misclassification. Third, the Lancet framework was operationalised using anthropometry, laboratory measures, blood pressure, and disease history alone. Direct adiposity measures such as DXA, bioelectrical impedance, CT, or MRI were unavailable, and data on activity limitation, exercise tolerance, and quality of life were not collected. The Lancet Commission obesity categories used here should therefore be regarded as a surveillance-based operationalisation rather than a comprehensive clinical diagnosis. Fourth, complete-case analysis was used, and selection bias related to non-random missingness cannot be fully excluded. Fifth, the sample was restricted to urban adults from Haidian District, Beijing; generalisability to rural areas or other regions requires further evaluation.

## Conclusion

In summary, applying the Lancet Commission obesity framework to urban Chinese adults identified a high burden of clinical obesity and a small preclinical obesity group. BMI categories showed substantial discordance with Lancet obesity categories, indicating that BMI alone does not define obesity-related disease states. Metabolic impairment and hypertension were the main contributors to clinical obesity classification. These findings support the use of excess-adiposity and complication-based assessment as a complement to BMI in chronic disease surveillance.

## Data Availability

The datasets presented in this article are not readily available due to the sensitive nature of the research. Requests to access the datasets should be directed to Zhenyu Liu, liuzhenyu@mail.bjhd.gov.cn.
