# Supplementary material for: The lancet commission obesity categories, excess adiposity, and BMI discordance in urban Chinese adults: a cross-sectional survey in Beijing
Source: Front Nutr. 2026 Aug 4;13:1883408. doi: 10.3389/fnut.2026.1883408 (PMC13481167; doi:10.3389/fnut.2026.1883408)
Supplement: Supplementary file 1 [file Table_1.docx]

**Supplementary Material**

**Supplementary Tables**

**Table S1. Baseline characteristics of participants by age group.**

| **Characteristic** | **18–39 years** | **40–59 years** | **60–79 years** | **P value** |
| --- | --- | --- | --- | --- |
| Number (%) | 3,287 (48.7) | 3,139 (33.4) | 1,704 (18.0) |  |
| Age, years | 29.1 ± 6.2 | 49.3 ± 5.7 | 67.1 ± 5.0 | <0.001 |
| Sex |  |  |  | 0.001 |
| Men | 1,663 (50.8) | 1,638 (49.9) | 915 (46.2) |  |
| Women | 1,624 (49.2) | 1,501 (50.1) | 789 (53.8) |  |
| Marital status |  |  |  | <0.001 |
| Unmarried | 1,512 (53.6) | 239 (7.0) | 166 (9.3) |  |
| Married | 1,775 (46.4) | 2,900 (93.0) | 1,538 (90.7) |  |
| Education level |  |  |  | <0.001 |
| Primary school or below | 51 (1.1) | 275 (8.4) | 172 (10.0) |  |
| Junior high school | 495 (13.0) | 911 (27.5) | 526 (29.7) |  |
| Senior high school | 615 (18.1) | 724 (23.1) | 554 (36.1) |  |
| College or above | 2,126 (67.8) | 1,229 (40.9) | 452 (24.2) |  |
| Smoking status |  |  |  | <0.001 |
| nonsmoker | 2,448 (75.2) | 2,226 (72.5) | 1,276 (78.1) |  |
| former smoker | 61 (1.9) | 85 (2.4) | 119 (5.9) |  |
| current smoker | 778 (22.9) | 828 (25.1) | 309 (16.0) |  |
| Excessive alcohol consumption |  |  |  | <0.001 |
| No | 3,124 (95.5) | 2,874 (92.0) | 1,565 (93.1) |  |
| Yes | 163 (4.5) | 265 (8.0) | 139 (6.9) |  |
| Low physical activity |  |  |  | <0.001 |
| No | 2,225 (68.8) | 2,435 (78.1) | 1,380 (79.3) |  |
| Yes | 1,062 (31.2) | 704 (21.9) | 324 (20.7) |  |
| Insufficient fruit and vegetable intake |  |  |  | <0.001 |
| No | 1,787 (52.8) | 1,898 (60.3) | 1,231 (74.0) |  |
| Yes | 1,500 (47.2) | 1,241 (39.7) | 473 (26.0) |  |
| Excess red meat intake |  |  |  | <0.001 |
| No | 2,122 (63.8) | 2,316 (73.0) | 1,327 (77.8) |  |
| Yes | 1,165 (36.2) | 823 (27.0) | 377 (22.2) |  |
| Height, cm | 168.2 ± 8.6 | 164.8 ± 8.2 | 162.5 ± 7.8 | <0.001 |
| Weight, kg | 70.9 ± 16.7 | 69.5 ± 13.0 | 67.1 ± 10.8 | <0.001 |
| BMI, kg/m² | 24.9 ± 4.7 | 25.5 ± 3.6 | 25.4 ± 3.4 | <0.001 |
| Waist circumference, cm | 82.7 ± 13.1 | 85.8 ± 11.0 | 87.7 ± 9.9 | <0.001 |
| WHtR | 0.5 ± 0.1 | 0.5 ± 0.1 | 0.5 ± 0.1 | <0.001 |
| Total sleep duration |  |  |  | <0.001 |
| ≤7 h | 299 (9.3) | 459 (14.4) | 271 (15.4) |  |
| 7~9 h | 1,571 (46.7) | 1,617 (52.3) | 814 (49.0) |  |
| ≥9 h | 1,417 (44.0) | 1,063 (33.3) | 619 (35.6) |  |
| Insomnia |  |  |  | <0.001 |
| No | 3,095 (93.8) | 2,870 (91.2) | 1,473 (85.7) |  |
| Yes | 192 (6.2) | 269 (8.8) | 231 (14.3) |  |
| BMI category |  |  |  | <0.001 |
| Underweight | 157 (5.0) | 46 (1.3) | 28 (2.0) |  |
| Normal | 1,365 (43.3) | 1,078 (34.5) | 621 (33.4) |  |
| Overweight | 987 (28.4) | 1,280 (41.9) | 716 (42.3) |  |
| Obesity | 778 (23.2) | 735 (22.3) | 339 (22.2) |  |
| Lancet obesity category |  |  |  | <0.001 |
| No obesity | 2,098 (66.7) | 1,670 (54.6) | 791 (45.1) |  |
| Preclinical obesity | 165 (5.1) | 109 (3.3) | 30 (2.0) |  |
| Clinical obesity | 1,024 (28.2) | 1,360 (42.1) | 883 (52.9) |  |
| **Diagnostic components for clinical obesity** |  |  |  |  |
| Metabolic impairment |  |  |  | <0.001 |
| No | 1,365 (42.9) | 1,012 (32.2) | 365 (19.2) |  |
| Yes | 1,922 (57.1) | 2,127 (67.8) | 1,339 (80.8) |  |
| Hyperglycemia |  |  |  | <0.001 |
| No | 2,879 (89.1) | 2,174 (69.4) | 775 (42.4) |  |
| Yes | 408 (10.9) | 965 (30.6) | 929 (57.6) |  |
| Hypertriglyceridemia |  |  |  | <0.001 |
| No | 2,279 (71.0) | 1,813 (58.2) | 909 (52.1) |  |
| Yes | 1,008 (29.0) | 1,326 (41.8) | 795 (47.9) |  |
| Low HDL-C |  |  |  | 0.029 |
| No | 1,872 (56.8) | 1,899 (59.8) | 1,085 (62.6) |  |
| Yes | 1,415 (43.2) | 1,240 (40.2) | 619 (37.4) |  |
| Hypertension |  |  |  | <0.001 |
| No | 2,779 (85.5) | 2,084 (66.8) | 742 (43.7) |  |
| Yes | 508 (14.5) | 1,055 (33.2) | 962 (56.3) |  |
| Chronic kidney disease |  |  |  | <0.001 |
| No | 3,032 (92.2) | 2,790 (88.8) | 1,328 (78.0) |  |
| Yes | 255 (7.8) | 349 (11.2) | 376 (22.0) |  |
| Chronic respiratory disease |  |  |  | <0.001 |
| No | 3,227 (98.3) | 3,034 (96.6) | 1,574 (90.7) |  |
| Yes | 60 (1.7) | 105 (3.4) | 130 (9.3) |  |
| Musculoskeletal disease |  |  |  | <0.001 |
| No | 2,867 (88.5) | 2,316 (73.4) | 1,032 (59.2) |  |
| Yes | 420 (11.5) | 823 (26.6) | 672 (40.8) |  |
| Coronary heart disease |  |  |  | <0.001 |
| No | 3,282 (99.9) | 3,086 (98.3) | 1,545 (91.8) |  |
| Yes | 5 (0.1) | 53 (1.7) | 159 (8.2) |  |
| Atrial fibrillation |  |  |  | <0.001 |
| No | 3,285 (100.0) | 3,117 (99.0) | 1,657 (97.0) |  |
| Yes | 2 (0.0) | 22 (1.0) | 47 (3.0) |  |
| **BP and biochemical parameters** |  |  |  |  |
| SBP, mm Hg | 120.5 ± 16.3 | 127.8 ± 18.0 | 136.5 ± 17.4 | <0.001 |
| DBP, mm Hg | 74.7 ± 11.5 | 79.7 ± 11.8 | 76.5 ± 10.3 | <0.001 |
| Fasting glucose, mmol/L | 5.2 ± 1.5 | 5.6 ± 1.8 | 6.0 ± 1.6 | <0.001 |
| HbA1c, % | 5.5 ± 0.8 | 5.9 ± 1.0 | 6.2 ± 1.0 | <0.001 |
| Total cholesterol, mmol/L | 4.7 ± 0.9 | 5.0 ± 1.0 | 5.0 ± 1.2 | <0.001 |
| Triglycerides, mmol/L | 1.6 ± 1.9 | 1.9 ± 2.0 | 1.7 ± 1.0 | <0.001 |
| HDL-C, mmol/L | 1.2 ± 0.3 | 1.2 ± 0.3 | 1.3 ± 0.3 | 0.001 |
| LDL-C, mmol/L | 2.8 ± 0.7 | 3.1 ± 0.8 | 3.0 ± 0.9 | <0.001 |
| UACR, mg/g | 4.0 (0.1, 6.3) | 4.4 (0.1, 8.0) | 5.1 (0.1, 11.3) | <0.001 |
| eGFR, mL/min/1.73 m² | 114.5 ± 17.8 | 103.0 ± 11.4 | 88.6 ± 12.9 | <0.001 |

*Values are presented as unweighted number (weighted %), weighted mean ± weighted SD, or median (IQR). P values were obtained from survey-weighted linear regression (continuous variables) or Rao-Scott χ² tests (categorical variables) comparing across age groups. BMI, body mass index; WHtR, waist-to-height ratio; SBP, systolic blood pressure; DBP, diastolic blood pressure; HbA1c, glycated haemoglobin; HDL-C, high-density lipoprotein cholesterol; LDL-C, low-density lipoprotein cholesterol; UACR, urinary albumin-to-creatinine ratio; eGFR, estimated glomerular filtration rate; ISI, Insomnia Severity Index.*

**Table S2. Cross-tabulation of BMI categories and Lancet obesity categories.**

| **Variable** | **Normal weight (BMI <24.0kg/m²)** | **Overweight (BMI 24.0–27.9kg/m²)** | **Obesity (BMI ≥28.0kg/m²)** | **P value** |
| --- | --- | --- | --- | --- |
| Total | 3,295 (41.8) | 2,983 (35.4) | 1,852 (22.8) |  |
| **Age group, years** |  |  |  | **<0.001** |
| 18–39 | 1,522 (48.4) | 987 (28.4) | 778 (23.2) |  |
| 40–59 | 1,124 (35.7) | 1,280 (41.9) | 735 (22.3) |  |
| 60–79 | 649 (35.5) | 716 (42.3) | 339 (22.2) |  |
| **Sex** |  |  |  | **<0.001** |
| Men | 1,306 (32.1) | 1,732 (39.9) | 1,178 (28.1) |  |
| Women | 1,989 (51.5) | 1,251 (31.0) | 674 (17.5) |  |
| **Lancet obesity category** |  |  |  | **<0.001** |
| No obesity (no excess adiposity) | 3,047 (93.4) | 1,469 (53.5) | 43 (3.4) |  |
| Preclinical obesity | 31 (1.2) | 152 (4.9) | 121 (7.5) |  |
| Clinical obesity | 217 (5.3) | 1,362 (41.6) | 1,688 (89.1) |  |

*Values are presented as unweighted number (weighted %). P values in section rows were calculated using Rao-Scott chi-square tests for global differences across BMI categories.*

*BMI, body mass index.*

**Table S3. Age- and sex-specific distribution of no obesity, preclinical obesity, and clinical obesity under the Lancet algorithm.**

| **Variable** | **No obesity (no excess adiposity)** | **Preclinical obesity** | **Clinical obesity** | **P value** |
| --- | --- | --- | --- | --- |
| Total | 4,559 (58.8) | 304 (3.9) | 3,267 (37.3) |  |
| **Age group, years** |  |  |  | **<0.001** |
| 18–39 | 2,098 (66.7) | 165 (5.1) | 1,024 (28.2) |  |
| 40–59 | 1,670 (54.6) | 109 (3.3) | 1,360 (42.1) |  |
| 60–79 | 791 (45.1) | 30 (2.0) | 883 (52.9) |  |
| **Sex** |  |  |  | **<0.001** |
| Men | 1,927 (48.9) | 211 (5.3) | 2,078 (45.8) |  |
| Women | 2,632 (68.6) | 93 (2.6) | 1,189 (28.8) |  |

*Values are presented as unweighted number (weighted %). P values in section rows were calculated using Rao-Scott chi-square tests for global differences in the distribution of Lancet obesity categories across age groups and by sex.*

**Table S4. Assessment of the proportional odds assumption.**

| **Predictor** | **OR (95% CI) — Cut 1** | **OR (95% CI) — Cut 2** | **\|ΔlogOR\|** |
| --- | --- | --- | --- |
| Age group: 40–59 (vs 18–39) | 1.48 (1.25–1.75) | 1.58 (1.33–1.87) | 0.065 |
| Age group: 60–79 (vs 18–39) | 2.32 (1.77–3.04) | 2.60 (2.01–3.35) | 0.114 |
| Sex: Women (vs Men) | 0.44 (0.38–0.51) | 0.50 (0.42–0.59) | 0.128 |
| Education: Junior high (vs Primary or below) | 1.27 (0.98–1.65) | 1.22 (0.94–1.59) | 0.04 |
| Education: Senior high (vs Primary or below) | 0.94 (0.74–1.18) | 0.92 (0.72–1.18) | 0.022 |
| Education: College or above (vs Primary or below) | 0.95 (0.78–1.16) | 0.91 (0.73–1.13) | 0.043 |
| Marital status: Married (vs Unmarried) | 1.22 (1.03–1.43) | 1.25 (1.08–1.45) | 0.024 |
| Smoking: Former (vs Never) | 0.80 (0.57–1.14) | 0.85 (0.59–1.22) | 0.061 |
| Smoking: Current (vs Never) | 1.10 (0.96–1.25) | 1.22 (1.05–1.43) | 0.104 |
| Excessive alcohol: Yes (vs No) | 1.17 (0.91–1.51) | 1.23 (0.93–1.62) | 0.05 |
| Low physical activity: Yes (vs No) | 0.99 (0.88–1.12) | 0.98 (0.86–1.12) | 0.01 |
| Insufficient fruit/vegetable intake: Yes (vs No) | 0.91 (0.79–1.04) | 0.88 (0.77–1.01) | 0.034 |
| Excess red meat intake: Yes (vs No) | 1.03 (0.91–1.17) | 0.97 (0.85–1.09) | 0.06 |
| Sleep duration: ≤7 h (vs 7–9 h) | 1.09 (0.88–1.35) | 1.06 (0.88–1.28) | 0.028 |
| Sleep duration: ≥9 h (vs 7–9 h) | 0.91 (0.80–1.03) | 0.86 (0.76–0.97) | 0.057 |
| Insomnia: Yes (vs No) | 0.81 (0.63–1.04) | 0.84 (0.66–1.07) | 0.036 |

*Cut 1: no obesity versus preclinical obesity/clinical obesity. Cut 2: no obesity/preclinical obesity versus clinical obesity. The proportional odds assumption was considered tenable when |ΔlogOR| ≤0.2; values marked * indicate |ΔlogOR| >0.4, suggesting potential violation.*

**Supplementary Figures**

**Supplementary Figure S1. Operational algorithm for classifying excess adiposity, preclinical obesity, and clinical obesity according to the Lancet Commission framework. Excess adiposity was confirmed when at least one anthropometric criterion was met; descriptive components of excess adiposity were assigned according to the mutually exclusive priority order described in the Methods. BMI, body mass index; WHtR, waist-to-height ratio; WC, waist circumference.**
